# Supplementary material for: FoodOmicsGR_RI: A Consortium for Comprehensive Molecular Characterisation of Food Products
Source: Metabolites. 2021 Jan 27;11(2):74. doi: 10.3390/metabo11020074 (PMC7911248; doi:10.3390/metabo11020074)
Supplement: Supplementary file 1 [file metabolites-11-00074-s001.pdf]

Supplementary Materials

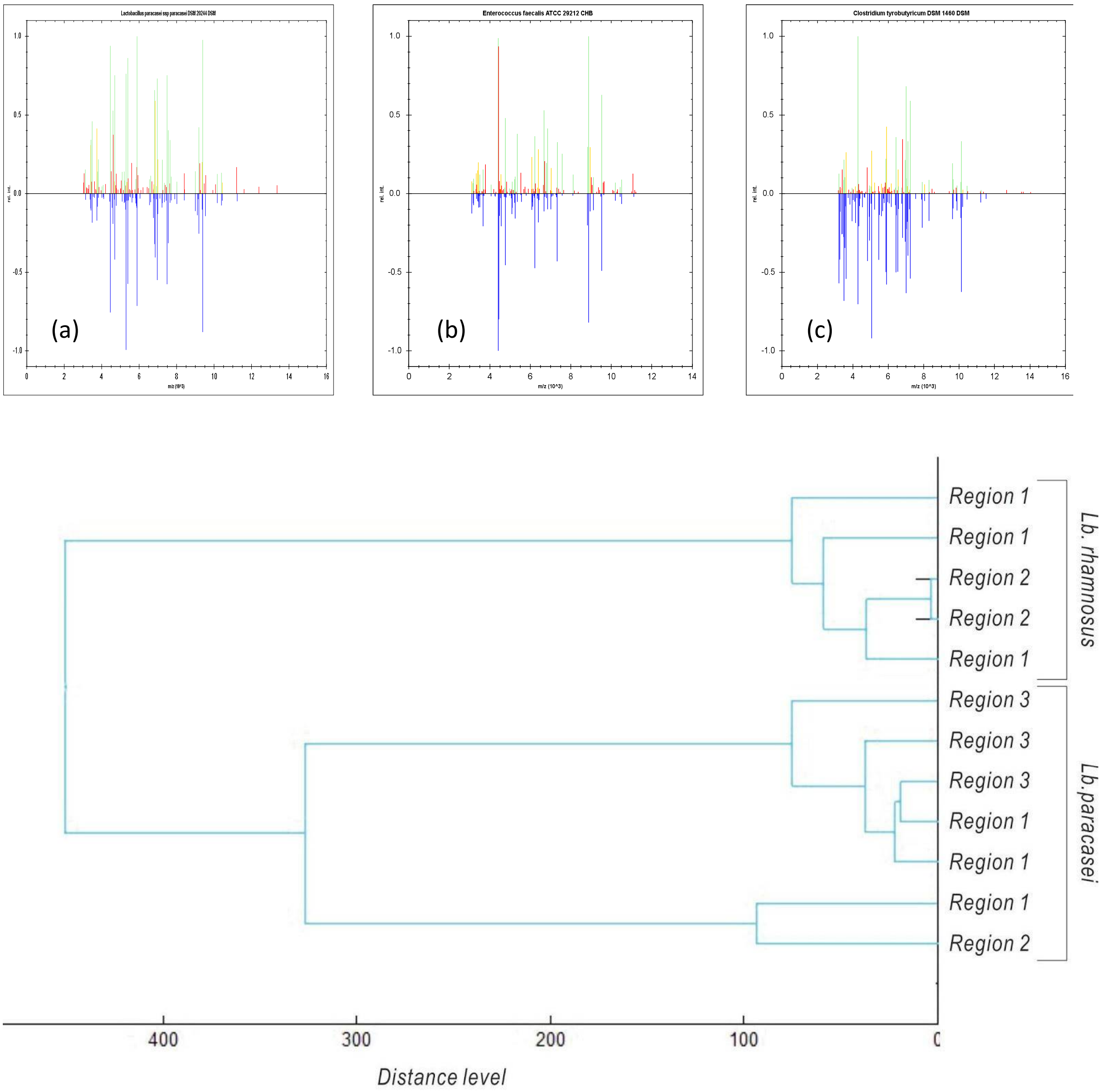

Figure S1. A characteristic Example of cheese classification based on MALDI-TOF-MS analysis. (**a**): Spectrum pattern: *Lactobacillus paracasei* (beneficial microorganism) (**b**): Spectrum pattern: *Enterococcus faecalis* (potentially pathogenic microorganism) (**c**): Spectrum pattern: *Clostridium tyrobutyricum* (spoilage microorganism). Every strain was compared to library's reference spectrum pattern (blue peaks) and the similarity of the examined strain was visualised by the green peaks. (**d**): Phyloproteomic tree (dendrogram) of *Lactobacillus rhamnosus* and *Lactobacillus paracasei* revealing the bacterial diversity according to the milk origin (Regions 1–3). The genetic diversity and geographical stability among the identified strains was evaluated by the spectrum fingerprinting profile, which was analysed and used to construct an Main Spectra Profile (MSP) dendrogram, using the standard Bruker software package (**d**).

The microbial community was studied with culture-independent methods, based on DNA extraction that offered the possibility of profiling uncultivable members of the microbial community, as well as distinguishing those that are metabolically active. DNA extraction was performed in cheese samples and the genomic material was isolated, by combining mechanical (beat beating) and chemical lysis of the cell. The purified DNA was processed in high through-put sequencing techniques, giving insights into cheese microflora composition and exhibiting unique bacterial profiles which varied in diversity and abundance of taxa. Although the cheeses were visually similar (white color and softness, texture), their bacterial profiles were very different at nearly every classification level. In this purpose, after the DNA extraction, the V3–V4 region of 16S rRNA genes was amplified using universal primers for bacteria and a classification of 16S rRNA targeted amplicon reads was performed using a taxonomic database. The collected data provided interactive visualizations and raw classification outputs per sample

FoodOmics Structure

Structure of the Facility

In particular the Aristotle University (AUTh) contributes in FoodOmicsGR_RI with the Departments of Chemistry (Laboratories of Analytical Chemistry, Food Technology, Environmental Science), the Dept. of Agricultural Sciences (Pomology, Enology, Plant Growth, Apiculture research groups and laboratories), Dept. of Veterinary Science (Animal Husbandry, Histology Laboratories), Dept. of Medicine (Toxicology Research group), Dept. of Informatics (Databases Laboratory), Dept. of Pharmacy (Pharmacognosy), Dept. of Sports Science (Physical Exercise Biochemistry and Nutrition Laboratory) and Dept. of Biology (Animal and Plant Genetics research groups). In total 16 staff members and more than eight new researchers with strong activity on food analysis and bioanalysis, metabolomics, biochemistry, bio-informatics and the genetic and molecular characterization of foods are working together. The core bioanalytical AUTh team is a recognized metabolomics group with pioneering work on method development-validation of metabolomics analytical tools, untargeted and targeted metabolic profiling methods, metabolomics approaches for the classification of foods (fruits, wine, and others) and the study of nutrition related disorders, and multi-analyte methods for the control of foods. The Auth teams are strongly linked and interconnected and have numerous common research projects, co-supervision of PhD and MSc studies and smaller scale research projects.

The University of Athens (UoA) contributes with the Departments of Chemistry (Analytical Chemistry and Food Chemistry laboratories) and the department of Pharmacy (NMR spectroscopy center). The University brings in unique sample sets, expertise in the analysis of foods by High Resolution Mass Spectrometry, and NMR spectroscopy, identification of bioactive compounds, and determination of contaminants. They have long experience in working with the food industry and participation in food-related projects at national and international level and have pioneered the development of metabolic profiling research in Greece.

University of Crete (UoC) contributes with the Department of Chemistry (NMR spectroscopy center). UoC team specializes in the application of NMR metabolomics in food analysis and authentication. UoC has developed protocols for quality control, specializing in the phenolic profiling of olive oil, wine, cheese, beverages. They also developed metabolomics approaches for the authentication of indigenous products (Cretan gravieure, PDO wines), nutrition clinical studies in support of health claims from functional foods.

The Agricultural University of Athens (AUA) is leading the elemental analysis (ICP-MS center) in food and biological samples. The team is working on Food Authentication through Synchronous Fluorescence spectroscopy, high throughput automated methods based on clinical chemistry principles transferred to the agri-food sector and elemental metabolomics linking environmental, food, nutrition and health sciences.

The Biomedical Research Center of the Academy of Athens (Center for Systems Biology) is leading the proteomics analysis. The group has expertise on proteomics and the analysis of protein function with numerous publications and patents in the field of foodomics/nutriomics.

The University of Ioannina (UoI) is leading food safety research (Dept. Chemistry) and the study and characterization of dairy products (Dept. of Agricultural Sciences). The groups have long experience in lactic acid bacteria isolation, identification of probiotics, microbiota analysis in farm animals, analysis of protein structure and function, proteomics on milk/cheese production from indigenous goat. The Chemistry Department groups (Analytical Chemistry and Food Chemistry) are expert groups on the analysis of pollutants, chemotherapeutants and contaminants with emphasis on food safety assessment and the characterization of bioactive, antioxidant and/or xenobiotic compounds in foods.

The University of the Aegean leads the research on the use and exploitation of food waste from industrial processes (Dept. of Environmental Sciences). The group has expertise on agro-industrial-municipal wastewater characterization/treatment, investigating the cultivation of aquatic plants with wastewater for the production of biomass with high content of proteins and starch, the recovery of antioxidant phenolic compounds from olive mill wastewater and the detection of micropollutants.

The International University (Dept. of Nutritional sciences) leads a “horizontal” effort on the assessment and prioritization of research actions and the establishment of food content databases. The group brings expertise in Food Biochemistry and Nutrition Sciences.

Table S1. List of MS metabolomics protocols

	Technology	Approach	Instrument	Mode	Specimen/ Study	Sample Prep	Data Treatment	Statistics	Reference
1	LC QTOF MS	Untargeted LC-MS polars	UPLC- QTOF-MS Premier/Tims-TOF	HILIC, ESI, full scan MS	foods, biological fluids, cell culture	LLE, protein precipitation	XCMS	PCA, PLS-DA, HCA, AUC ROC	119
2		Untargeted LC-MS lipophilic		RPLC, ESI, full scan MS	foods, biological fluids, cell culture	LLE, protein precipitation	XCMS		
3		Targeted - bioactive content/antioxidants	UPLC- QTOF-MS	RPLC, ESI negative, bbCID	olive oil	LLE (MeOH:H2O, 80:20 v/v)	Target screening of 43 antioxidants	quantitative analysis - regression analysis	6
4		Untargeted bioactive content	UPLC- QTOF-MS	RPLC, ESI, bbCID	olive oil	LLE (MeOH:H2O, 80:20 v/v)	suspect screening of 1600 suspect compounds	PCA, PLS-DA, ACO-RF	6
5		Untargeted - bioactive content	UPLC- QTOF-MS	RPLC, ESI, bbCID + Auto MS (for identification)	olive oil	LLE (MeOH:H2O, 80:20 v/v)	XCMS, CAMERA, IPO	PCA, PLS-DA, ACO-RF	6
6		Targeted - bioactive content/antioxidants	UPLC- QTOF-MS	RPLC, ESI negative, bbCID	fruit juices	direct injection	Target screening of 37 antioxidants	quantitative analysis - regression analysis	9
7		Untargeted - bioactive content	UPLC- QTOF-MS	RPLC, ESI, bbCID + Auto MS (for identification)	fruit juices	direct injection	XCMS, CAMERA, IPO	differential analysis, PCA, PLS-DA	9

8		Untargeted - bioactive content	UPLC- QTOF-MS	RPLC, ESI, bbCID + Auto MS (for identification)	table olives	LLE (MeOH:H2O, 80:20 v/v)	XCMS, CAMERA, IPO	differential analysis, PCA, PLS-DA	30
9	LC QqQ MS (MRM)	Targeted HILIC-MS/MS	UPLC-QqQ, Xevo TqD	HILIC Amide, pos/neg ESI	Honey/Royal jelly	dilution	Target Lynx, MRM 120 analytes		53, 52
10		Targeted HILIC-MS/MS			Carobs	LLE			
11		Targeted HILIC-MS/MS			Nutrition (blood, urine, feces, tissue liver kidney brain etc)	LLE			52-54
12		Targeted HILIC-MS/MS			various in vitro studies HEPG2, NPCs…				
13		Targeted HILIC-MS/MS	UPLC-QqQ, Thermo Access		milk and wine		Xcalibur, MRM 80 analytes		
14		HILIC-MS/MS	UPLC-QqQ, Xevo TqD		AminoAcid Panel urine	dilution	Target Lynx, MRM 45 analytes		
15		RPLC-MS/MS	UPLC-QqQ, Sciex 3200/6500+	C8 ESI	Bile Acid Panel blood	LLE	MRM 18 analytes		In preparation
16		RPLC-MS/MS	UPLC-QqQ, Xevo TqD	C18 ESI	Carnitines blood	LLE	Target Lynx, MRM 16 analytes		

17		RPLC-MS/MS	UPLC-QqQ, Xevo TqD	C18 ESI MRM	pharmaceuticals in biological samples	LLE	Target Lynx, MRM 84 analytes	regression analysis	157
18		RPLC-MS/MS	UPLC-QqQ, Sciex 3200	C18 ESI MRM	Ceramides in blood	LLE	MultiQuant, MRM 4 analytes		Begou el at in preparation
19		RPLC-MS/MS		C18 ESI	25OH VitaminD2 /D3 blood	LLE/SLE	MultiQuant MRM 4 analytes		in preparation
20	GC MS(MS)	GC-MS	GC-Q Agilent 5973	EI, full scan	small molecules untargeted	MeOX, MSTFA derivatisation	GAVIN, AMDIS, NIST, Fiehn Agilent & Maurer Spectral Databases	PCA, PLS-DA, HCA, AUC ROC	47, 118
21		GC-MS	GC- EVOQ TQ	EI, full scan	VOCs in biological samples	HS-SMPE, HS			
22		GC-MS		EI, full scan	VOCs in foods (olive oil, spirits)	HS-SMPE, HS			
23		GC-MS/MS		EI MRM MS	small molecules targeted, saliva/blood	MeOX, MSTFA derivatisation	quantitative 12 acids	regression analysis	
24		GC-MS/MS		EI MRM-MS	Organic Acids urine	MeOX, MSTFA derivatisation	quantitative 54 acids C3-C18		Mouskeftara et al in preparation
25		GC-MS/MS / GC-FID		EI-MRM-MS	Fatty Acids blood	Methyl esterification	quantitative, 34 acids C12-C24		Mouskeftara et al submitted

Table S2. List of NMR metabolomics protocols.

	Analysis Approach	Aim	Instrument MHz	Mode	Food/Study	sample Prep	Statistics / Data Treatment	Reference
1	NMR, Untargeted	Polar metabolite and lipid profiling	NMR 600/500	liquid state 1H	biological fluids (blood, urine), feces extracts, cell culture, olive, olive oil, wine, honey, natural products and extracts	none	PCA, OPLS-DA, OPLS	64, 122
2	NMR, Untargeted	Polar metabolite profiling	NMR 300, 500	liquid state 1H	liquid foods (wine, honey, juice, coffee, milk etc.)	none	PCA, OPLS-DA, OPLS	58
3	QNMR, Targeted	Phenolic profiling	NMR 500	liquid state 1H, 31P	wine, olive oil, fruits, plants, etc	freeze drying, SPE	PCA, OPLS-DA, OPLS	59, 60, 52
4	NMR, Untargeted	Polar metabolite profiling	NMR 300, 500	liquid state 1H	solid foods (cheese, coffee, plants)	freeze drying, LE	PCA, OPLS-DA, OPLS	64
5	NMR, Untargeted	Lipid profiling	NMR 300, 500	liquid state 1H	solid foods (cheese, coffee, etc)	freeze drying, LE	PCA, OPLS-DA, OPLS	64
6	QNMR, Targeted	Small molecule compositional analysis	NMR 300, 500	liquid state 1H	foods, plants	depending on sample	-	11
7	NMR, Untargeted	Compositional analysis	NMR 400	solid state 1H, 13C	meat, cheese, plants, fruits, etc	none	PCA, OPLS-DA, OPLS	12
8	NMR, Untargeted	Polar metabolite and lipid profiling	NMR 400	solid state 1H, 13C	tissues, cell culture	none	PCA, OPLS-DA, OPLS	

Table S3. List of Genetics-Genomics protocols.

	Aim	specimen	Platform	Method	Instrument Platform	Reference
1	Genetic identification of species in food products	meat, fish, seafood, game	DNA barcoding	gene DNA sequencing	PCR, DNA Sequencer	73
2	Traceability of animal species in food products	meat products	Real time PCR	proportional DNA amplification and quantification	rt-PCR	
3	Species milk identification	sheep milk, goat milk	Casein dinstiction	gene DNA sequencing	PCR, DNA Sequencer	
4	Origin traceability of wild and hatchery individuals	sea bream, sea bass	SNP panels	SNP genotyping (Kasp)	rt-PCR	74, 75
5	Genomic characterization of livestock species	livestock species	SNP arrays	GWAS analysis	Illumina NGS	76
6	Microbiome analysis	Cheese	NGS	16S rRNA sequencing	Illumina NGS	93
7	Strain Typing	Cheese	Ribo Printing	rRNA gene restriction pattern analysis	DuPont Ribo Printer	

Table S4. List of proteomics protocols.

	Analysis	Technology	Instrument	Food/Study	Sample Prep	Data Treatment	Statistics	Reference
1	Proteomics	High Resolution Mass Spectrometry	nanoLC-MS/MS Orbitrap Elite	Dairy Products	Protein extraction/Tryptic digestion (GR1005463, GR1009574)	Total proteins	Unique Proteins	77
2	Peptidomics	High Resolution Mass Spectrometry	nanoLC-MS/MS Orbitrap Elite	Dairy Products	Peptide extraction ((GR1005304, GR1005463, EPA1748297, GR1005463)	Natural Ocurring Peptides and Small Proteins	Unique Peptides/Small proteins	77
3	Proteomics	High Resolution Mass Spectrometry	nanoLC-MS/MS Orbitrap Elite	Wine	Protein extraction/Tryptic digestion (GR1005463, EPA1748297)	Total proteins	Unique Proteins	
4	Peptidomics	High Resolution Mass Spectrometry	nanoLC-MS/MS Orbitrap Elite	Wine	Peptide extraction (GR1005304, GR1005463, EPA1748297, GR1005463)	Natural Ocurring Peptides and Small Proteins	Unique Peptides/Small proteins	
5	Proteomics	High Resolution Mass Spectrometry	nanoLC-MS/MS Orbitrap Elite	Honey	Protein extraction/Tryptic digestion (GR1005463, EPA1748297)	Total proteins	Unique Proteins	
6	Peptidomics	High Resolution Mass Spectrometry	nanoLC-MS/MS Orbitrap Elite	Honey	Peptide extraction (GR1005304, GR1005463, EPA1748297, GR1005463)	Natural Ocurring Peptides and Small Proteins	Unique Peptides/Small proteins	
7	proteomics analysis of bacterial strains	MALDI-TOF MS	Bruker LT Microflex	Cheese microbiome analysis				

Table S5. List of MS elemental metabolomics protocols.

Technique	Instrument	Sample Preparation	Analytes	reference
Inductively Coupled Plasma - Mass Spectrometry	Perkin Elmer ELAN 9000	microwave assisted digestion	Rare Earth Elements - Ultra trace elements	82–86

Table S6. List of food safety assessment protocols.

.	Instrumentation	Sample preparation Method	Sample	Purpose	reference
1	UHPLC-LTQ Orbitrap MS	QuEChERS	Honey	Pesticide Residues	
2	UHPLC-LTQ Orbitrap MS/ GC-MS	QuEChERS	Oil	Pesticide Residues	140
3	UHPLC-LTQ Orbitrap MS	QuEChERS	Wine	Pesticide Residues	144
4	UHPLC-LTQ Orbitrap MS	QuEChERS	Milk	Pesticide Residues	143
5	UPLC-MS/MS (QTOF or QqQ)	LLE/SPE	foods	Determination of NIAS in Foods and food contact materials	150
6	GC-MS/MS	LLE/SPE	foods	Determination of endocrine disruptors in Foods	151
7	LC-MS/MS	LLE/SPE	foods	Determination of endocrine disruptors (including Bisphenol) in Foods	146
8	RPLC-MS/MS	SLE	Fish tissue, porcine, poultry meat	Vetetinary drug residues (17 sulfonamides and 5 tetracyclines)	152
9	RPLC-MS/MS	SLE/UAE	Milk powder, butter, fish tissue and eggs	Veterinary drugs and pharmaceutical residues (115 compounds)	153
10	UHPLC-QTOF MS	fish: SLE/UAE milk: LLE/SPE	Milk and fish tissue	Veterinary drugs and pharmaceutical residues (143 compounds)	154
11	HILIC-MS/MS	SLE	Bovine muscle tissue	Veterinary pharmaceuticals (76 compounds, 13 classes)	155
12	HILIC-MS/MS	SLE/dSPE	Animal tissues and eggs	Coccidiostats (16 compounds)	156

Table S7. List of food authenticity/traceability RIS priorities that are covered by FoodOmicsGR_RI work.

	**Relevant RIS3 Priorities Related to Food Characterization and Development of New Products**
1	Improving the competitive position of Greek food products from agricultural, livestock and aquaculture products in international markets
2	Enhancement and improvement of characteristics of Greek products of primary production with an emphasis on quality and safety, 3
3	Utilization of "omics" technologies for the genetic/molecular characterization with the scope of assessing and improving domestic agricultural biodiversity (vegetables, herbs, wild plants, bees, animals, breeds, fish, etc) and their consolidation as Greek product
4	Development of methods and technologies for the control of authenticity (authentication) of domestic agricultural products, identification and/or certification of geographic origin with modern techniques
5	Highlighting and improving the unique characteristics of Greek processed products with emphasis on quality and product safety
6	Improve the quality, durability and safety of foods, including aquaculture products, beverages and feed by the development and use of improved control methodologies
7	Development and use of fast and low cost non-destructive and non-invasive methods for the control and detection of biological, chemical and physical hazards (such as persistent organic contaminants, heavy metals, perfluorinated compounds and toxins) in foods and in the food chain
8	Development of novel products containing bioactive ingredients from the Greek flora and marine organisms (e.g. natural colours, antioxidants, sweeteners)
9	Development of methods for the recovery of these components and their integration into the final product avoiding the loss of their biological properties
10	Analysis of Greek foods with emphasis on micro-nutrients, molecular analysis and characterization of domestic genotypes (varieties) with interesting commercial characteristics and Complement Greek Food databases

Table S8. List of food nutritional value RIS priorities that are covered by FoodOmicsGR_RI work.

	**Relevant Nutrition health and wellness related RIS3 priorities**
1	Enhancing the understanding of the relationship between diet, health and wellbeing in relation to agricultural food products and ingredients
2	Assessment of the nutritional value of Greek food
3	The role of traditional Greek nutrition and indigenous foods in the enhancement of the health of the Greek population
4	Creating food products with reduced concentration in undesired food ingredients either for the whole population or food for consumers with special dietary requirements, such as allergens, gluten etc
5	Investigation of the relationship between nutrition, health and wellness: Consumer issues, socially, culturally, technologically as well as traditional aspects of food, including issues related to nutritional disorders, with the aim to develop foods and diets appropriate to meet the particular needs of special consumer groups (people with allergies, pregnant women, infants, children, elderly, etc
6	Study of genetic variations in the metabolism of essential nutrients (Nutrigenetics), and the role of nutrients in gene expression (Nutrigenomics)
7	Recording and promoting health claims of Greek products with an emphasis on traditional products
